# Association of various risk factors with prognosis and hospitalization cost in Chinese patients with acute myocardial infarction: A clinical analysis of 627 cases

**DOI:** 10.3892/etm.2014.2087

**Published:** 2014-11-24

**Authors:** PEINING WANG, BIN ZHANG, LIJUN JIN, HONGTAO LIAO, TAIMING DONG

**Affiliations:** Department of Cardiology, Guangdong Cardiovascular Institute, Guangdong Academy of Medical Science, Guangdong General Hospital, Guangzhou, Guangdong 510100, P.R. China

**Keywords:** acute myocardial infarction, prognosis, hospitalization cost, risk factors, Chinese population

## Abstract

Acute myocardial infarction (AMI) is the leading cause of morbidity and mortality in the developed world and is becoming increasingly more common in developing countries. The risk factors affecting the prognosis of Chinese patients may differ from those in other populations. This study was conducted to investigate the potential risk factors that may correlate with prognosis and hospitalization costs of Chinese AMI patients. A total of 627 hospitalized AMI patients were recruited and their general information and relevant laboratory parameters were collected. Accordingly, the patients were grouped into different subgroups and potential risk factors and their correlations with prognosis and hospitalization costs were analyzed. Age, high blood pressure, infarct location and percutaneous coronary intervention (PCI) were the variables significantly associated with the differences in the prognosis of AMI patients (P<0.05), whereas times and duration of hospitalization, high blood pressure, infarct location and PCI treatment were found to be significantly associated with the cost of hospitalization (P<0.05). However, the AMI patients enrolled in this study may not be representative of all AMI patients in China. In addition, the prognosis of these patients was limited to their hospital stay. Therefore, long-term follow-up requires careful assessment.

## Introduction

Acute myocardial infarction (AMI) is one of the most frequently occurring cardiovascular diseases and the leading cause of morbidity and mortality in the developed as well as the developing world (1). Over 7.5 million individuals reportedly succumb to AMI annually worldwide (2), with United States alone accounting for >600,000 deaths annually (3). Although formerly considered an illness of predominantly developed countries, myocardial infarction (MI) is becoming increasingly more common in developing countries; although its occurrence was reported to be lower compared to that in Western populations (4), the prevalence and mortality rates have been increasing rapidly. Epidemiological statistics in 2003 reported >700,000 new cases annually in China and this increasing trend has attracted widespread attention (5).

The research on the pathogenesis, treatment and prognosis of AMI has progressed significantly over the last decade. However, the underlying mechanism remains unclear, although the risk of AMI has been suggested to be associated with exposure to multiple risk factors (6). A large international case-control study of risk factors for MI conducted in 52 countries (the INTERHEART study) reported 9 easily measured and potentially modifiable risk factors, among which 4 were associated with lifestyle and accounted for 54.6% of the population attributable risk. The remaining 5 factors were high blood pressure, diabetes, abdominal obesity, social psychological factors and lipid levels (7). A recent study also demonstrated evidence suggesting that the prognosis of AMI may be associated with risk factors including myocardial ischemia, infarction times and locations, demographic data such as age and gender and complications such as high blood pressure and diabetes (8).

The effect of these 9 risk factors was suggested to be consistent across all geographic regions and ethnic groups worldwide and the management of AMI was considered one of the most significant advances of contemporary cardiology, with reduced mortality reported by several registries (9). However, the prevalence of these risk factors varies among different populations (7,10) and the availability of data on the risk factors, management and outcome of AMI in developing countries was limited. The majority of the registries that documented the use of various medications and interventional procedures in AMI patients were established in developed countries and the majority of available data from developing countries regarding AMI management were derived from small studies from single centers.

Being the world’s largest developing country, China reportedly accounts for >700,000 deaths annually (11). With an aging population and a projected substantial rise in disease rate, the burden of AMI in China has increased significantly. However, there was no clear concept of the factors associated with the prognosis of AMI among Chinese patients and large population-based studies on the potential risk factors and prognosis of AMI are scarce. The prevalence and strength of the association of risk factors observed in other populations may not necessarily apply to China, due to the genetic susceptibility and environmental factors. In this study, the potential risk factors and prognosis of AMI were investigated among a large Chinese population and the correlations of AMI risk factors with prognosis and hospitalization cost were analyzed.

## Patients and methods

### Subjects

A total of 627 AMI patients who were admitted to our hospital between January, 2009 and December, 2012 were recruited in this study. The inclusion criteria were as follows: i) Patients presented with clinical symptoms of AMI, including pain radiation, abdominal pain, chest tightness, chest pain, dizziness and nausea; ii) dynamic changes in the electrocardiogram (ECG), such as ST segment elevation and depression, T wave inversion, the presence of any pathological Q wave and abnormal R wave; iii) increasing myocardial damage; and iv) all the patients were in the acute phase of MI.

### Study design

This study was approved by the local Ethics Review Board of our institution and all the patients and their family members provided written informed consent prior to inclusion. General information, such as gender, age, complications (hypotension, high cholesterol and hyperlipidemia), infarct location, times of hospitalization, percutaneous coronary intervention (PCI), hospitalization time and cost, disease diagnosis, treatment mode and prognosis, was collected. The data were entered into Excel software and a database was set up for statistical analysis.

### Grouping

According to the different classification criteria, the patients were divided into different subgroups. All the cases were primarily divided into the death and non-death groups and the latter was further subdivided into healed, improved, and non-healed subgroups, according to the prognosis of the patients. Based on the ECG diagnosis of the infarct location, the patients were grouped into anterior wall, anteroseptal, inferior wall, posterior wall, right ventricle, high lateral wall and multiple-wall MI. Moreover, the patients were further classified according to times of hospitalization (1, 2, and ≥3), age (≤53, 54–68 and ≥69 years) and duration of hospitalization (≤6 and >6 days).

### Statistical analysis

Statistical analysis was performed using GraphPad Prism 6.0 software (GraphPad Software, La Jolla, CA, USA). Count data are presented as % of total number and measurement data are expressed as mean ± standard deviation (SD). Comparisons between the groups were performed with the χ^2^ test. Data of normal distribution and homogeneity of variance between the two groups were compared using analysis of variance and independent samples t-test and the remaining data were analyzed using non-parametric test. P<0.05 was considered to indicate statistically significant differences.

## Results

### Basic patient information

A total of 627 AMI patients, aged 26–90 years (mean age ± SD, 60.56±11.66 years), including 549 men and 78 women, were recruited in this study. The patients’ general information was tabulated and is presented in Table I. A total of 279 patients (44.50%) had high blood pressure, 146 (23.29%) had a history of diabetes and 27 (4.31%) had hyperlipidemia. As regards infarct location, 267 patients were diagnosed with anterior wall AMI, accounting for 42.58% of all AMI patients, followed by inferior wall AMI (42.11%) and anteroseptal, posterior wall, multiple-wall, high lateral wall and right ventricular AMI (9.41, 1.75, 1.75, 1.28 and 1.12%, respectively). Among all patients, 556 (88.7%) ultimately underwent PCI treatment. The majority of the patients (566/627) were recorded as being hospitalized for the first time (90.27%), 42 (6.70%) for the second time and 19 (3.03%) for the third time or more. The hospitalization time ranged between 0 and 58 days, with an average of 6.74±5.68 days. The cost of hospitalization ranged from 1,639 up to 299,184 Yuan, with an average cost of 57,594±37,396 Yuan.

### Comparison of relevant factors between death and non-death groups

**Figure 1:**
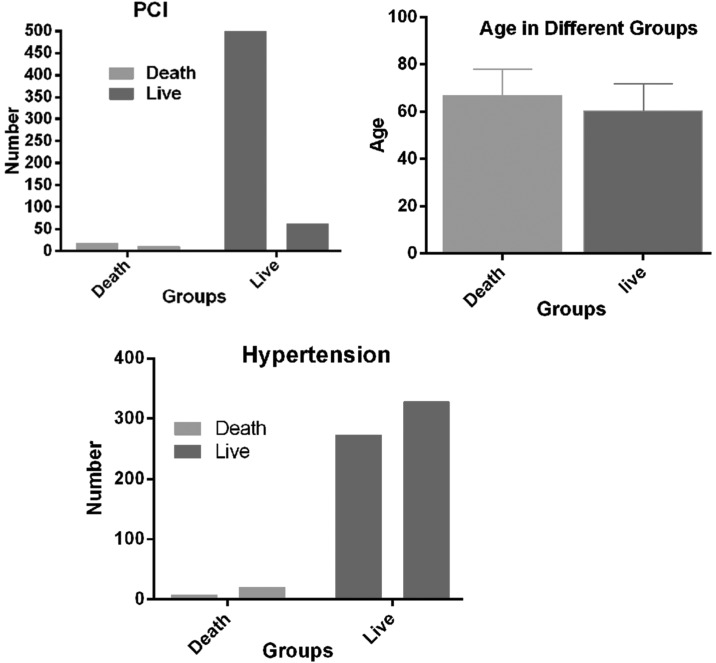
The prognosis of acute myocardial infarction patients was shown to be significantly correlated with age, hypertension prevalence and the number of patients undergoing percutaneous coronary intervention (PCI) treatment.

As presented in Table II, 27 patients succumbed during follow-up (aged 39–89 years). The hospitalization time ranged between 0 and 26 days and the hospitalization cost was estimated to be between 2,731 and 298,100 Yuan. There were 600 patients in the non-death group, aged 26–90 years, with a hospitalization time of 1–58 days. The hospitalization cost ranged between 1,639 and 299,184 Yuan. There were significant age differences between the death and non-death groups (P=0.0039), with the age of patients in the death group being significantly higher compared to that of the non-death group. The final status of the patients was also found to be significantly correlated with high blood pressure (P=0.047) and PCI treatment (P<0.001). The proportion of patients suffering from high blood pressure was significantly lower in the death group compared to that in non-death group, while the number of patients undergoing PCI treatment was significantly higher in the non-death group. By contrast, there were no significant differences in hospitalization time, hospitalization cost, gender, infarct location, times of hospitalization, diabetes and hyperlipidemia between the two groups (P>0.05; Table II and Fig. 1).

### Single-factor analysis of relevant factors in different prognostic groups

**Figure 2:**
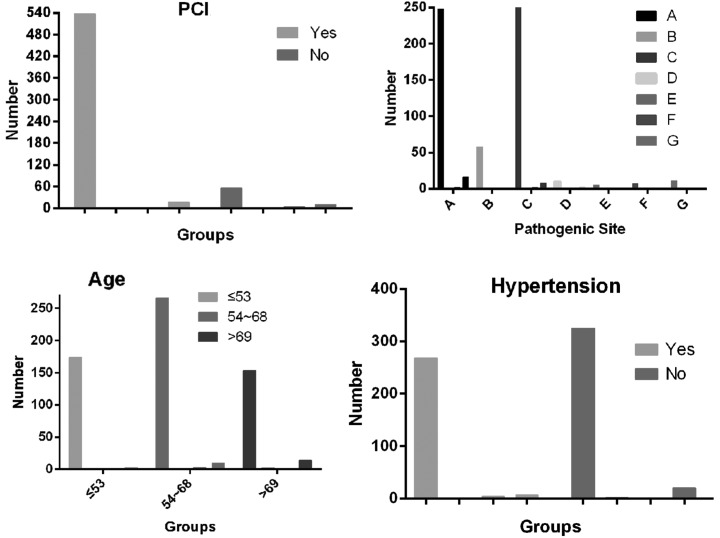
Single-factor analysis of relevant factors in different prognostic groups. The prognosis of acute myocardial infarction (AMI) patients was positively correlated with percutaneous coronary intervention (PCI) treatment and negatively with age distribution and incidence of hypertension (P<0.05). The proportion of AMIs located in the inferior and anterior wall predominated in both the death and non-death groups. Infarct location: A, anterior wall; B anteroseptal; C inferior wall; D posterior wall; E right ventricle; F high lateral wall; G multiple-wall.

The results of the statistical analysis revealed that relevant factors, including age (P=0.015), high blood pressure (P=0.045), infarct location (P<0.0001) and PCI treatment (P<0.0001), were significantly associated with prognosis, while gender, hospitalization time, diabetes, hyperlipidemia and times of hospitalization did not exhibit a strong correlation with prognosis (P>0.05; Table III and Fig. 2).

### Analysis of factors affecting hospitalization cost

**Figure 3:**
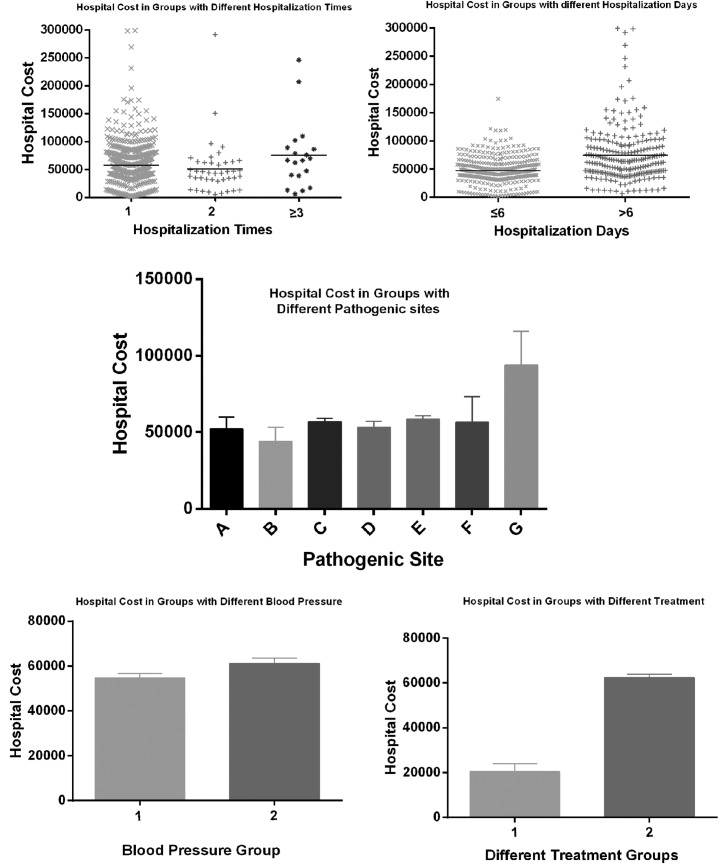
Single-factor analysis of correlation between hospitalization cost and risk factors. The statistical analysis demonstrated that times and duration of hospitalization (P=0.045 and P<0.0001, respectively), high blood pressure (P=0.029), infarct location (P=0.046) and number of patients udergoing percutaneous coronary intervention (P<0.0001) were significantly associated with hospitalization cost. Infarct location: A, anterior wall; B, anteroseptal; C inferior wall; D, posterior wall; E, right ventricle; F high lateral wall; G multiple-wall.

According to the analysis results mentioned above, the correlation of hospitalization costs with other relevant factors was further investigated. The results demonstrated that times of hospitalization (P=0.045), duration of hospitalization (P<0.0001), high blood pressure (P=0.029), infarct location (P=0.046) and PIC (P<0.0001) were significantly associated with hospitalization cost, while there was no obvious correlation between hospitalization cost and gender, age, prognosis, hyperglycemia or hyperlipidemia (Fig. 3).

## Discussion

MI has long been recognized as a complication of grave prognosis. The characteristics of the patients hospitalized with AMI have reportedly changed over the past 3 decades in several aspects (8). A large population-based study on the 9 potential risk factors of AMI and their correlation with prognosis and hospitalization cost was systematically performed among the Chinese population. In comparison to other studies, our study was unique in providing detailed data regarding potential risk factors of AMI among Chinese individuals and their correlations with prognosis and hospitalization cost.

The findings of the present study demonstrated that age, high blood pressure, infarct location and PCI were the factors significantly associated with differences in the prognosis of AMI (P<0.05), while there was no significant correlation between prognosis and gender, hospitalization cost, times and duration of hospitalization. Furthermore, the correlation analysis of hospitalization cost with relevant factors revealed a significant association of hospitalization cost with high blood pressure, infarct location, PCI, times and duration of hospitalization among the study groups, whereas factors including gender, age, prognosis, hyperglycemia and hyperlipidemia, were not found to be strongly correlated with hospitalization cost.

Age has long been recognized as one of the significant factors affecting the prognosis of AMI. It was reported that >50% AMI cases in the United States were patients aged ≥65 years and 80% of the patients who succumbed to AMI were aged ≥65 years (12). Individuals aged ≥75 years, constituting 6.1% of the US population, accounted for 36% of AMI cases and 60% of deaths (13). Although old age *per se* does not necessarily predispose an individual to disease, certain factors arising from age-related physical, cognitive and social circumstances may contribute to a higher susceptibility. In addition, with increasing age, the possibility of multivascular disease, high blood pressure and other complications also increases significantly and exacerbates the poor prognosis (14,15).

Recent trends revealed a shift in the age distribution of AMI patients with an increase in AMIs occurring in patients aged ≥85 years, with the mean age increasing from 78.0 to 80.1 years (16), while a younger trend regarding the onset of AMI was also observed (17), with the underlying reason remaining unknown. In this study, the subjects were classified into 4 groups according to their prognosis and age distribution. Significant differences were demonstrated regarding age and prognosis, which was consistent with previous results. However, there was no significant age difference between the death and non-death groups. The possible causes of this phenomenon are as follows: i) There was a limited number of cases in the death group, which may result in inadequate statistical power; ii) the non-death group was further subgrouped and the difference between sample levels may fail to reflect whether they were combined, resulting in mixture samples; and iii) only representative samples from our hospital were included, which may result in certain limitations.

A previous study in Framingham over 26 years demonstrated that the mortality rate from coronary heart disease among men (60%) was twice that of women (18). Accordingly, the incidence of female AMI was also lower compared to the incidence in males, but the mortality rate of AMI was comparatively higher in female patients (19). Unfortunately, we were unable to reach the same conclusion, due to the limited number of female patients recruited.

The main mechanism underlying AMI was hypothesized to induce acute thrombosis on the basis of coronary atherosclerotic plaque rupture, with almost complete obstruction of the coronary artery completely (20). The location of the infarct was found to be an important prognostic indicator that warrants consideration in the stratification of risk and clinical management of MI patients (21). The prognosis of patients with anterior MI was reported to be significantly worse compared to that of inferior MI; in addition, anterior wall infarction was suggested to be associated with more extensive myocardial damage compared to inferior wall infarction (22). Our results demonstrated that the proportion of AMIs with infarcts of inferior and anterior location predominated in both the death and non-death groups, which was consistent with the results of a previous study (23). A total of 59.3% of patients who succumbed to the disease had anterior and 29.6% had inferior wall AMI. Patients with anterior AMI exhibited a significantly higher mortality rate compared to those with inferior infarction, suggesting that anterior wall infarcts were more significantly associated with poor prognosis compared to inferior wall infarcts, corroborating the results reported by Strauss *et al* (24). The underlying reason remains unclear, but it may be associated with segmental contradiction movement, hemodynamic changes and the malignant arrhythmia of anterior wall AMI.

PCI is currently a focal point of AMI treatment in order to restore reperfusion of the infarct artery, rescue the dying myocardium and preserve cardiac function (16). A previous study demonstrated that the prognosis of AMI improved when patients underwent PCI, which reduced the occurrence of coronary artery occlusion and patient mortality (25). PCI may effectively prevent and reverse the left ventricular wall muscle remodeling following MI and improve left ventricular function. Multiple clinical prospective randomized comparative studies suggested that, if the patients with acute ST-elevation MI are transferred to an intervention center for direct PCI within 3 hours, the results may be superior to those of immediate thrombolysis treatment in the local hospital (26). In the present study, we found that 556 of the 627 AMI patients received PCI treatment during the hospitalization period, accounting for >88.7% of all hospitalized patients. PCI treatment reduced the mortality of AMI from 14.1 to 3.06%, with ~96% of the patients healing from AMI after receiving PCI. However, distal microvascular thrombotic obstruction and ventricular remodeling may occur following PCI (27) and additional treatment, such as anticoagulants and antiplatelet drugs, should be considered in combination with clinical emergency PCI. In addition, PCI may also lead to coronary spasm, perforation and acute occlusion; therefore, further testing is required to improve patient performance.

As the traditional atherosclerotic risk factors, diabetes, hypertension and dyslipidemia were considered to be independently associated with a higher risk of in-hospital mortality. It has been reported that crude mortality was higher among MI patients with diabetes (11.9%) and hypertension (9.8%) and lowest among patients with dyslipidemia (4.6%). However, these factors were not as significant in predicting hospital mortality following MI compared to other clinical factors, in contrast to the general expectations of the clinicians. The inclusion of atherosclerotic risk factors in models of hospital mortality did not improve the predictive ability beyond other major clinical and sociodemographic characteristics (28).

Hypertension is traditionally considered as an important contributing factor associated with AMI occurrence and prognosis (29). The INTERHEART study indicated that classical risk factors contribute differently to AMI risk in different populations (7). The prevalence of hypertension in Chinese AMI patients was reported to be 47%, while a marginally lower incidence of ~44.5% was detected in the present study. The results of our study also revealed that the incidence of hypertension was significantly different between the death and non-death groups and it was significantly correlated with the prognosis of AMI.

Diabetes mellitus is a well-established risk factor for cardiovascular diseases and its presence alone may increase the risk of cardiovascular events to the same extent as a previous episode of AMI. The AFIRMAR study reported that diabetes mellitus was independently associated with AMI in the Brazilian population (30). According to the INTERHEART study, a reported history of diabetes was found in 18% of AMI cases (7) and China alone accounted for 50% of the total number of diabetics worldwide. However, the prevalence of diabetes among Chinese AMI patients was comparable to that in Western countries (31). Diabetes has also been suggested to be one of the negative predictors of long-term outcome during hospitalization (32). However, our study demonstrated a similar incidence of diabetes between the death and non-death groups; in addition, there was no significant correlation between the occurrence of diabetes and the prognosis of AMI.

Moreover, a literature review revealed that the prevalence of hypercholesterolemia was variable among patients with AMI in different regions (33). Hypercholesterolemia was found to be a significant risk factor for AMI patients (34), with a direct association between the severity of hyperglycemia and the scope of MI and prognosis (35). However, a previous study on AMI in an Indian population also indicated that hypercholesterolemia was not a significant risk factor for AMI (6) and the same result, with no significant difference in the prognosis of AMI, was obtained by our study, regardless of whether the hypercholesterolemia was complicated. Further investigation is required to elucidate the significance of this finding.

The times and duration of hospitalization were also hypothesized to reflect the severity of the disease to some extent; hospitalization time was longer among survivors (7.46±4.01 days) compared to non-survivors (6.86±3.30) (36). However, no significant correlations of prognosis with times of hospitalization and duration of hospital stay were identified.

Our study demonstrated that times and duration of hospitalization, high blood pressure, infarct location and PIC were significantly associated with hospitalization cost. As previously mentioned, the duration of hospital stay reflects the severity of the disease, while other factors, including high blood pressure, infarct location and PCI treatment may also be associated with the severity of AMI, which inevitably affects the cost during the hospital stay. However, the pursuit of a higher income and overuse of PCI cannot be excluded.

Non-random samples are prone to bias. The AMI patients and hospitals enrolled may not be representative of all AMI patients and hospitals in China. In addition, although controlling for potential confounding factors, we cannot exclude the effect of other unmeasured factors, which may have affected prognosis and hospitalization costs for AMI. Finally, the prognosis of AMI patients was limited to their hospital stay and long-term follow-up requires careful assessment.

Our study presented evidence indicating that the risk factors affecting the prognosis of Chinese patients may differ from those in other countries. Age, infarct location, high blood pressure and PCI were significantly correlated with prognosis of AMI patients, whereas hospitalization cost was affected by high blood pressure, infarct location, PCI, times and duration of hospitalization.

## Figures and Tables

**Table I:** General information of patients with acute myocardial infarction (AMI).

Variables	Patient no. (n=627)	%
Age (years)		
Mean ± SD	60.56±11.66	
Range	26–90	
Male patients	549	87.56
Infarct location		
Anterior wall	267	42.58
Inferior wall	264	42.11
Anteroseptal	59	9.41
Posterior wall	11	1.75
Multiple-wall	11	1.75
High lateral wall	8	1.28
Right ventricle	7	1.12
Times of hospitalization		
1	566	90.27
2	42	6.70
≥3	19	3.03
Complications		
Diabetes mellitus	146	23.29
High blood pressure	279	44.50
Hyperlipidemia	27	4.31
Duration of hospitalization (days)		
Mean ± SD	6.74±5.68	
Range	0–58	
Number of patients receiving PCI	556	88.7
Hospitalization costs (Yuan)		
Mean ± SD	57,594±37,396	
Range	1,639–299,184	

SD, standard deviation; PCI, percutaneous coronary intervention.

**Table II:** Comparison of 8 death relevant factors between the death group and non-death group.

Relevant factors	Non-death group, no. (%)(n=600)	Death group, no. (%)(n=27)	P-value
Age (years), mean ± SD	60.28±0.47	65.89±2.14	0.0039^a^
Male patients	526	23	0.76
Infarct location		0.14	
Anterior wall	251 (41.8)	16 (59.3)	
Inferior wall	256 (42.7)	8 (29.6)	
Anteroseptal	59	(9.83) 0	
Posterior wall	10 (1.67)	1 (3.7)	
Multiple-wall	11 (1.83)	0	
High lateral wall	7 (1.17)	1 (3.7)	
Right ventricle	6 (1)	1 (3.7)	
Times of hospitalization		0.39	
1	543 (90.5)	23 (85.2)	
2	40 (6.67)	2 (7.4)	
≥3	17 (2.83)	2 (7.4)	
Complications		1.00	
Diabetes	140 (23.3)	6 (22.2)	
High blood pressure^a^	272 (45.3)	7 (25.9)	0.047^a^
Hyperlipidemia	26 (4.3)	1 (3.7)	1.00
Duration of hospitalization (days), mean ± SD	6.73±0.23	6.78±1.50	0.10
Number of patients receiving PCI	539 (89.8)	17 (63.0)	<0.0001^a^
Hospitalization costs (Yuan), mean ± SD	57,016±1,435	70,442±13,654	0.89

a P<0.05 was considered statistically significant.

SD, standard deviation; PCI, percutaneous coronary intervention.

**Table III:** Comparison of 9 relevant factors between different prognostic groups.

Factors affecting prognosis	Non-death group (n=600)			Death group (n=27)

	Healed (n=593)	Improved (n=2)	Not healed (n=5)	
Infarct location^a^				
Anterior wall	248/267	1/267	2/267	16/267
Anteroseptal	58/59	0/59	1/59	0/59
Inferior wall	254/264	0/264	2/264	8/264
Posterior wall	10/11	0/11	0/11	1/11
Right ventricle	5/7	1/7	0/7	1/7
High lateral wall	7/8	0/8	0/8	1/8
Multiple-wall	11/11	0/11	0/11	0/11
Gender				
Male	521/549	2/549	3/549	23/549
Female	72/78	0/78	2/78	4/78
Age (years)^a^				
≤53	174/178	0/178	1/178	3/178
54~68	266/279	0/279	3/279	10/279
≥69	153/170	2/170	1/170	14/170
Times of hospitalization				
1	537/566	2/566	4/566	23/566
2	40/42	0/42	0/42	2/42
≥3	16/19	0/19	1/19	2/19
Duration of hospitalization (days)				
≤6	370/392	2/392	3/392	17/392
>6	223/233	0/233	2/233	10/233
Diabetes mellitus				
Yes	138/146	0/146	2/146	6/146
No	455/481	2/481	3/481	21/481
High blood pressure^a^				
Yes	268/279	0/279	4/279	7/279
No	325/348	2/348	1/348	20/348
Hyperlipidemia				
Yes	26/27	0/27	0/27	1/27
No	567/600	2/600	5/600	26/600
PCI^a^				
Yes	538/556	0/556	1/556	17/556
No	55/71	2/71	4/71	10/71

a Significantly associated with prognosis.

PCI, percutaneous coronary intervention.
